# Dietary Protein Intake, Breast Feeding and Growth in Human Milk Fed Preterm Infants

**DOI:** 10.3390/ijerph15061196

**Published:** 2018-06-07

**Authors:** Emma Tonkin, Jacqueline Miller, Maria Makrides, Andrew J. McPhee, Scott A. Morris, Robert A. Gibson, Carmel T. Collins

**Affiliations:** 1 Nutrition and Dietetics, College of Nursing and Health Sciences, Flinders University, Adelaide, SA 5001, Australia; emma.tonkin@flinders.edu.au (E.T.); jacqueline.miller@sahmri.com (J.M.); 2 Healthy Mothers, Babies and Children, South Australian Health and Medical Research Institute, Adelaide, SA 5001, Australia; maria.makrides@sahmri.com (M.M.); andrew.mcphee@sa.gov.au (A.J.M.); robert.gibson@adelaide.edu.au (R.A.G.); 3 Adelaide Medical School, Discipline of Paediatrics, The University of Adelaide, Adelaide, SA 5001, Australia; 4 Neonatal Medicine, Women’s and Children’s Hospital, Adelaide, SA 5006, Australia; 5 Centre for Perinatal Medicine Flinders Medical Centre and School of Medicine, Flinders University, Adelaide, SA 5042, Australia; scott.morris@sa.gov.au; 6 School of Agriculture, Food and Wine, The University of Adelaide, Adelaide, SA 5064, Australia

**Keywords:** breast feeding, dietary proteins, enteral nutrition, infant–premature, milk–human, weight gain

## Abstract

Protein intakes of preterm infants are frequently below recommendations, but few studies report accurate intakes due to the difficulty of analysing human milk clinically. This observational analysis from a randomised trial of infants born <31 weeks’ gestation, investigating two levels of protein fortification, reports protein intakes compared with requirements and determines the association of direct breastfeeding on growth. Ninety-two infants (median gestational age 28 weeks, Interquartile range (IQR) 26–29; mean birth weight 1040 g, SD 300 g) were studied. Infants born weighing <1000 g were underfed protein compared with recommendations (median (IQR) intake of 3.0 (2.0–3.7) g/kg/day in week 2 versus recommendation of 4–4.5 g/kg/day), while those born weighing ≥1000 g met recommended protein intakes after the first week of life (median (IQR) intake of 3.7 (3.0–4.0) g/kg/day in week 2 versus recommendation of 3.5–4.5 g/kg/day). A moderate, negative correlation between the mean number of breast feeds and change in rate of weight gain (*r* = −0.37, *p* = 0.001) was found. Protein intakes of infants <1000 g did not meet recommendations and all infants were underfed protein and energy in the first week of life. Current protein fortification is inadequate for infants born <1000 g. Exploratory analysis showed faltering rate weight gain associated with increasing number of breast feeds and these results warrant confirmation.

## 1. Introduction

The importance of adequate nutrition for preterm infants to support growth is well known [1]. Better neurodevelopmental outcomes at 18 months are associated with improved rate of growth before, rather than after, 40 weeks post-menstrual age (PMA) [2,3] and the use of human milk [4]. Thus, optimising dietary management and nutrient intake to ensure quality postnatal growth before term equivalent is important [5]. Feeding preterm infants is complex, and evidence from contemporary dietary intake studies on preterm infants born <1500 g, fed with fortified human milk, suggest infants currently have energy intakes above recommendations [6,7] while receiving inadequate protein in the early weeks of life [7,8,9,10]. Yet few studies report accurate macronutrient intakes of human milk (HM) fed infants due to difficulty in assessing HM composition in the clinical setting [6,8]. Assumed values for the protein concentration of HM used in the literature vary from 12 g/L [9] to 15 g/L [8]. Thus studies comparing actual with assumed protein intakes can either under [8] or overestimate [9] actual intakes highlighting the importance of measuring HM composition.

HM is widely acknowledged as the preferred feed for preterm infants’ due to the immunological and nutritional benefits it confers [1,11,12]. However, HM requires fortification with protein, energy and micronutrients for infants born weighing <1500 g to meet nutritional requirements [13] with current typical commercial fortifiers providing an additional 0.8 to 1.8 g protein per 100 mL of expressed breast milk (EBM). The optimal level of protein fortification is still under debate but there is some evidence that protein fortification regimes are inadequate to meet protein requirements [8,14,15]. Transitioning from tube-feeding to oral intake via direct breast feeding is an important step in preparing preterm infants for discharge [16]. However, the potential to fortify feeds with key nutrients is lost with the introduction of direct breast feeds. While preterm infants post-discharge have been shown to adjust their volume of intake depending on calorie density [17] a Cochrane review of ad libitum feeding versus scheduled feeding for preterm infants during the neonatal admission has shown a lower nutrient intake in those infants on the ad lib regime [18]. Hence, human milk fed infants, while receiving many of the advantages that human milk confers, may have lower protein intakes during their neonatal admission because of the dual effect of inadequate fortification regimes during tube feeding and cessation of this fortification once direct breastfeeding commences. Additionally, mothers with inadequate breast milk supply will rely more on preterm formula during this interval and may therefore have higher protein intakes. 

A randomised trial evaluating the effect of a higher protein human milk fortifier (HMF) compared with standard protein, on preterm infant growth included analysis of mothers’ HM samples for protein and fat content, thus accurate enteral protein intakes for all infants could be reported [19]. Although all mothers involved in the trial intended to exclusively supply HM, a pragmatic approach was taken to feeding preterm formula when mother’s milk was insufficient. This is reflective of the mixed-feeding approach in clinical practice typical of the time, when few centres in Australia had human milk banks. Additionally, infants who developed serious illness, such as lung disease, were not excluded from participation. These data therefore provide the basis for the present study in which we aimed to: (1) describe actual protein and energy intakes of human milk fed preterm infants fed 2 different protein levels during their neonatal admission, stratified by birth weight (<1000 g and ≥1000 g) (2) to compare these with current recommended intakes and (3) to determine the association between direct breast feeding and growth. 

## 2. Materials and Methods 

### 2.1. Study Design and Participants

The original randomised trial [19] investigated the effect of a higher protein HMF on growth in preterm infants with the primary outcome of length gain. Participants were recruited from the Women’s and Children’s Hospital and Flinders Medical Centre (Adelaide, Australia) between October 2006 and June 2008. Study start was the date the study fortifier was introduced, and infants were followed until the estimated due date or discharge (whichever came first). Infants were included if they were born at <31 weeks’ gestation and their mother intended to supply human milk. Exclusion criteria included: major congenital or chromosomal abnormality, infants where extra dietary protein was contraindicated (e.g., phenylketonuria), low maternal milk supply and continuation of expressing HM uncertain and likely transfer to a hospital which would make follow up difficult. Ninety-two infants were enrolled in the original study and all were included in the present study. The details of recruitment, randomisation and blinding are reported elsewhere [19]. Ethics approval was granted by the human research ethics committee of the Children, Youth and Women’s Health Service (REC 1774) and Flinders Medical Centre (CDTC 187/067). 

### 2.2. Management of Feeds

Infants were randomly assigned to receive either the trial HMF (higher protein, containing 1.4 g protein added to 100 mL of HM) or control HMF (standard protein, containing 1 g protein added to 100 mL of HM). Energy was kept constant (17 kcal/100 mL) by the addition of carbohydrate to the standard protein fortifier. The composition of both HMFs are reported elsewhere [19]. Infants received parenteral nutrition for a short period immediately post-birth. When HM was unavailable due to insufficient supply, a standard preterm formula containing 2.4 g protein and 80 kcal per 100 mL was provided. A standardised enteral nutrition management protocol was used in both units as follows: Enteral feeds were commenced with unfortified expressed HM or if unavailable, preterm formula as soon as the infant was stable, aiming for day 1–2. Feeds were commenced at 5–10 mL/kg/day, advancing by 10–20 mL/kg/day as tolerated to a final volume of 160–180 mL/kg/day. HM fortification commenced when enteral intake reached ≥80 mL/kg/day.

### 2.3. Dietary Intake

Enteral intake data were collected prospectively from detailed clinical fluid balance charts. This included volume and caloric density of HM and formula, supplements provided and number of direct breast feeds. Enteral intake before enrolment and all parenteral intakes were collected retrospectively using fluid balance charts and drug orders. An aliquot of the infant’s pooled 24 h period unfortified EBM was analysed weekly and was assumed representative of the HM composition for ±3 days around the collection date. The sample comprised freshly expressed HM and/or thawed mother’s own milk when fresh HM was unavailable. Protein and fat analysis of the HM was done using mid-infrared spectroscopy (MilkoScan Minor™, Foss, Hillerød, Denmark). Lactose content of HM was not measured as it is the most stable nutrient in HM and assumed to be 6.8 g/100 mL [20]. The nutritional composition of fortifier, formula, parenteral nutrition solution and lipid solutions composition was as provided by the respective manufacturer. Energy intake was calculated using the Atwater factors of 4, 4 and 9 kcal per gram of protein, carbohydrate and fat, respectively. A direct breast feed was defined as any time an infant was put to the breast as noted in the fluid balance charts. No attempt was made to quantitate the amount of milk transfer during a breast feed. While it is acknowledged that nutritive sucking skills develop at different rates, [21] the volume of milk taken at a breast feed is minimal (<10 mL, approximately <5% of daily intake) before 34 weeks PMA [22]. We therefore considered direct breast feeds before 34 weeks PMA as non-nutritive and investigated the relationship between growth and direct breastfeeds after 34 weeks PMA and until discharge home or estimated due data (EDD). 

### 2.4. Anthropometry and Clinical Outcomes

Measurements were taken by trained staff using techniques outlined in the World Health Organization Multicentre Growth Reference Study [23]. Weight was measured at the same time daily in Intensive Care, and twice weekly in Special care using calibrated electronic balance scales accurate to 5 g. Length and head circumference were measured weekly to the nearest 1 mm using a recumbent length board (O’Leary; Ellard Instruments, Monroe, WA, USA) and paper tape respectively. Clinical outcomes were defined according to the Australian and New Zealand Neonatal Network [24] as follows: necrotising enterocolitis, Bell’s stage II or higher; chronic lung disease (CLD), oxygen requirement at 36 weeks post menstrual age, late-onset sepsis, positive blood culture and clinical signs. 

### 2.5. Statistical Methods

When a mother’s milk was insufficient for analysis the mean of that individual mother’s HM analyses was used to represent HM composition values for that week. Descriptive statistics are presented according to birth weight categories (<1000 g, ≥1000 g). Dietary intakes were compared with current recommendations [11,15,25]. The recommended nutrient intake levels reported in Koletzko et al. [25] are specific for birth weights up to 1500 g and provide recommendations for both parenteral and enteral nutrition and so are used as a comparison, noting that the most recent consensus guidelines by a panel of experts, recommending 3.5 to 4.5 g protein/kg [15] are encompassed within Koletzko. The relationship between direct breast feeding and growth was investigated using Spearman rank order correlation coefficient. A summary variable representing change in rate of weight gain after 34 weeks PMA for each individual infant was produced using linear regression [26,27] and the following equation:

Δm = m_2_ − m_1_ (1)

where Δm = Change in rate of weight gain (g/day), m_1_ = Rate of weight gain from 2nd day of regained birth weight to 34 weeks PMA (g/day) and m_2_ = Rate of weight gain after and including 34 weeks PMA (g/day) until study end (discharge or EDD).

Thus a negative change in rate of weight gain represents a slower rate of growth after 34 weeks PMA. The analysis was repeated after stratification for birth weight category and chronic lung disease (as determined by oxygen requirement at 36 weeks PMA). Infants were separated into groups according to their mean number of daily breast feeds after 34 weeks PMA (<1, 1–2, 2–3, ≥3) for descriptive analysis of change in growth. All statistical analyses were conducted using IBM SPSS Statistics version 19 (SPSS, Chicago, IL, USA). 

## 3. Results

### 3.1. Participant Characteristics and Clinical Outcomes

All 92 infants were followed to original study end and included in the present analysis. The characteristics of participating infants and their mothers are presented in Table 1. The clinical and growth outcomes of infants are presented in Table 2. A greater proportion of infants born <1000 g had chronic lung disease (*n* = 29 (66%) versus *n* = 8 (17%), confirmed necrotizing enterocolitis (*n* = 7 (16%) versus *n* = 1 (2%) and sepsis (*n* = 8 (18%) versus *n* = 5 (10%). Those infants born <1000 g however, had a slightly faster rate of fractional weight gain, defined as weight gain as a proportion of body weight in g/kg/day; (Mean, SD weight gain of 16.0, 1.5 versus 15.2, 2.1 g/kg/day in <1000 g and ≥1000 g birth weight, respectively) (Table 2). 

### 3.2. Nutritional Management

The randomised trial [19] analysis showed that nutritional management of the infants did not differ between groups except for protein intakes, we therefore present protein intake by birth weight (<1000 g and ≥1000 g) and by randomised group. Median daily volume and energy intakes between the randomised groups were not different [19] and are therefore presented according to birth weight category (<1000 g and ≥1000 g). Infants born <1000 g took longer to commence (Median (IQR) of 4 (3,5) versus 3, (2,4) days) and reach full enteral feeds (Median (IQR) of 19 (14,26) versus 13 (10, 17) days), and received parenteral nutrition for longer (Median (IQR) of 23 (16,30) versus 12 (8, 17) days) than infants born weighing ≥1000 g (Table 2). First week protein intakes did not reach recommendations regardless of birth weight category or to which protein fortifier group the infants were randomised (Table 3). Overall, infants born <1000g were underfed protein, regardless of protein fortifier group, while those born ≥1000 g met recommendations (Table 3).

### 3.3. Direct Breast Feeding and Growth

A moderate, negative correlation between the mean number of direct breast feeds and change in rate of weight gain after 34 weeks PMA (*r* = −0.37, *p* = 0.001) was found. Thus, the higher the number of direct breast feeds per day, the more the growth rate slowed after 34 weeks. This was more pronounced in infants born weighing ≥1000 g (*r* = −0.40, *p* = 0.005) and less so in infants born <1000 g (*r* = −0.24, *p* = 0.11). This was also the case in infants without chronic lung disease (*r* = −0.37, *p* = 0.005), compared with infants with chronic lung diseases (*r* = −0.20, *p* = 0.24). The characteristics of the groups according to the number of daily breastfeeds after 34 weeks are presented in Tables S1 and S2. Within each group, there were a similar number of infants allocated to protein fortifier groups. There were no infants with chronic lung disease or born <28 weeks’ GA in the breastfeed group with >3 breastfeeds/day. The lowest breastfeed group (<1 breastfeed/day) had a much lower proportion of their enteral intake as human milk, indicating that they consumed more preterm formula. 

**Figure 1:**
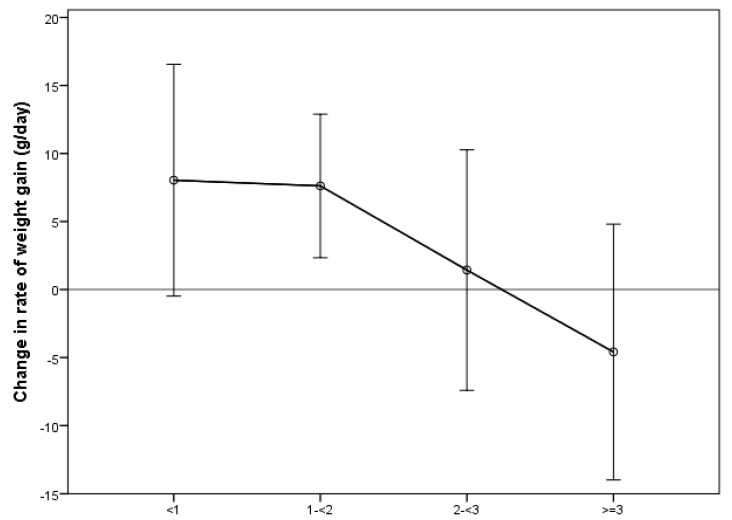
Mean change in rate of weight gain after 34 weeks for infants having <1, 1–<2, 2–<3 and ≥3 breast feeds per day. Markers represent mean for group, bars represent 1 standard deviation from mean. Solid line at zero indicates no change in rate of weight gain between growth periods.

The rate of weight gain for infants with a mean number of breastfeeds <1 per day after 34 weeks PMA increased by an average of 8.0 (SD 8.8) g/day (Figure 1), while infants with mean >3 breast feeds per day displayed faltering growth after 34 weeks PMA (mean change in rate of weight gain −4.6 g/day, SD 9.4 g/day). 

## 4. Discussion

In this study of infants born at <31 weeks gestation we found infants born ≥1000 g achieved protein intakes within recommendations and adequate energy intakes after the first week of life, while infants born <1000 g received insufficient protein, regardless of which protein fortifier they were allocated, and did not reach recommended energy intakes until week 3. Infants born <1000 g typically present the most complexity with regard to metabolic balance when feeding, thus it is not surprising they were underfed protein. This finding is also consistent with other studies showing inadequate protein intakes in these infants, particularly in the first weeks of life [7,8,10,29,30]. There is conflicting evidence regarding energy intakes however, with some studies showing recommendations are typically met [7,8] and others showing inadequate energy intakes consistent with our finding [10,29,30]. All infants were underfed protein and energy in the first days of life, with intakes barely reaching half the recommended intake by the third day of life. With studies showing protein intakes during this period to be an important predictor of growth [31,32] it is perhaps no surprise these infants only just reached intrauterine growth rates [28]. The long term implications of such intakes are also concerning, with first week protein intakes suggested to be predictive of neurodevelopmental outcome at 18 months [33]. A more aggressive approach to both parenteral and early enteral feeding may therefore be required.

In this exploratory analysis we have shown that the rate of weight gain slows as the number of daily breastfeeds increases. The group of infants having the highest number of direct breast feeds each day slowed their rate of weight gain by 4.6 g/day after the introduction of direct breast feeds. This is in contrast to those infants with <1 and 1–2 breast feeds per day, whose rate of growth increased by 8 and 7.6 g/day respectively. Growth rates increase after 34 weeks PMA as infants are generally more clinically stable and experience fewer interruptions to feeding. Growth percentile data for Australian preterm infants reported by Beeby et al. [34] when calculated in the same manner as in the present study, indicate an increased rate of growth of 3.9 g/day after 34 weeks as compared to before. Extrapolating the present results to a difference in weight at estimated due date, infants with the highest number of direct breast feeds would weigh ~190 g more if they maintained their previous rate of growth, and ~530 g more if they increased their rate of growth in the same manner as the infants with the lowest number of direct breast feeds. Of note however, the number of infants with mean >3 direct breast feeds per day was small (*n* = 9), and the majority of these infants were born ≥1000 g, which may explain the result found after stratification by birth weight category, as there may not be enough infants born <1000 g taking frequent direct breast feeds to examine the relationship in this group alone. Nonetheless, the clinical significance of this effect is substantial and warrants further, dedicated research to confirm the effect found in this small exploratory analysis.

There are a number of possible explanations for the faltering growth seen with increasing number of direct breast feeds and the cause is likely to be multifactorial. Firstly, the infants’ ability to self-regulate may not be developed sufficiently to increase the volume of intake and compensate for the lower caloric density of a breastfeed. Secondly, the infants with the lowest number of breastfeeds were those consuming more preterm formula, with a higher protein and energy content than human milk. Finally, it’s possible that the infants did not receive sufficient fat rich hind milk. Regardless of the reason, the slowed weight gain is of clinical concern. The psychological and attachment benefits of direct breast feeding for mother and infant are well documented [12,35]. It has also been shown that earlier initiation of, and therefore longer experience and practice with direct breast feeding results in earlier establishment of full breast feeding [22,36,37]. The achievement of sucking feeds is a crucial factor influencing readiness for discharge home in preterm infants [16,22]. Thus while frequent direct breast feeding appears to facilitate many desirable outcomes for preterm infants and their families [12] it may also compromise the goal of achieving optimal nutrient intake and growth during admission. Strategies to improve growth may include test weighing of breastfeeds to determine the volume taken more accurately, simply being mindful to not overestimate the volume taken or providing fortifier as a separate, concentrated solution before or after a direct breast feed. Infants may then gain the benefit of both early direct breast feeding and HM fortification, rather than have these as competing interests. These strategies may result in the preservation of the earlier growth rate and therefore better growth outcomes at discharge and warrants further investigation. 

The small number of infants taking a large number of direct breast feeds toward study end limited statistical analysis and thus strength of conclusions drawn from this study. That there were few infants born <1000 g having frequent direct breast feeds compared with infants born ≥1000 g additionally complicates interpretation of these results. However, the regular measurement of HM composition is a major strength of our study compared with many other studies which simply assume values of HM composition. The inclusion of infants with morbidities typical of infants born at <31 weeks gestation [38] ensures generalisability of the study to all infants treated in Australian neonatal intensive care units. Additionally, the pragmatic approach to feeding is reflective of Australian clinical practice, thus these results are highly relevant to the clinical setting.

## 5. Conclusions

Protein intakes of infants with birth weight <1000 g did not meet recommendations and all infants were underfed protein and energy in the first week of life. Greater attention to protein and energy intakes in the first week of life, and throughout the neonatal admission for the smallest babies i.e., those born <1000 g, deserve consideration. Faltering rate of weight gain was shown to be correlated with increasing number of direct breast feeds, and this warrants further research to confirm this observed effect.

## Figures and Tables

**Table 1:** Clinical characteristics of participants.

Infants (*n* = 92)	Whole Cohort (*n* = 92)	<1000 g (*n* = 44)	>1000 g (*n* = 48)
Gestational age (weeks)	28 (26–29)	26 (25–28)	29 (28–29)
Recruitment hospital			
Women’s and Children’s Hospital	80 (87)	38 (86)	42 (88)
Flinders Medical Centre	12 (13)	6 (14)	6 (13)
Male infants	40 (44)	19 (43)	21 (44)
Birth anthropometry			
Weight (g)	1036, 301	788, 156	1262, 209
Length (cm)	35.7, 3.5	32.9, 2.7 *	38.1, 1.9
Head circumference (cm)	25.4, 2.2	23.8, 1.8	26.9, 1.5
Caesarean section	59 (64)	31 (71)	28 (58)
Multiple births (number of infants)	24 (26)	10 (23)	14, (29)
Mothers	(*n* = 80)	(*n* = 39)	(*n* = 41)
Age (years)	30.2, 5.8	30.7, 6.2	29.1, 5.2
Smoked during pregnancy	15 (19)	8 (21)	7 (17)
Previous preterm birth	11 (14)	1 (3)	10 (24)
Received antenatal steroids	71 (89)	35 (90)	36 (88)
Received IVF	11 (14)	4 (10)	7 (17)
Completed Secondary Education ^†^	57 (72)	30 (77)	27 (66)

Data are presented as median (interquartile range), *n* (%), Mean, SD. * *n* = 43 infants; ^†^ *n* = 79 mothers; Abbreviations: IVF, in vitro fertilisation.

**Table 2:** Infant outcomes.

Clinical Outcomes	Whole Cohort (*n* = 92)	<1000 g (*n* = 44)	≥1000 g (*n* = 48)
Chronic lung disease ^†^	37 (40)	29 (66)	8 (17)
Received corticosteroid treatment	31 (34)	23 (53)	8 (17)
Confirmed necrotizing enterocolitis	8 (9)	7 (16)	1 (2)
Intraventricular haemorrhage ^‡^	19 (21)	9 (21)	10 (21)
Sepsis	13 (14)	8 (18)	5 (10)
Nasal CPAP	26 (28)	9 (21)	17 (35)
Length of stay ^§^	75.1, 18.6	87.1, 13.8	64.2, 15.4
Post-menstrual age at discharge or EDD (weeks)	38 (37–40)	39 (38–40)	37 (37–39)
Growth outcomes			
Days to regain birth weight, mean (SD)	11.2, 3.9	11.3, 4.3	11.0, 3.5
Weight gain (g/day) *	27.4, 5.7	25.0, 4.7	29.6, 5.8
Intrauterine weight gain (g/kg/day) [28]	15.0		
Weight gain (g/kg/day)	15.6, 1.8	16.0, 1.5	15.2, 2.1
Weight gain before 34 weeks PMA (g/day)	24.6, 7.3	21.2, 6.1	27.6, 7.0
Weight gain after 34 weeks PMA (g/day)	30.2, 6.5	29.5, 5.4	30.9, 7.4
Length gain (cm/week)	1.0, 0.1	1.0, 0.1	1.0, 0.2
Head circumference gain (cm/week)	0.9, 0.1	0.9, 0.1	0.9, 0.2
Discharge weight (g)	2697, 357	2662, 428	2729, 276
Dietary Outcomes			
Age study fortifier commenced (days)	13 (10–18)	15 (12–19)	11 (8–14)
Age enteral feeds commenced (days)	3 (2, 4)	4 (3, 5)	3 (2, 4)
Days to reach full enteral feeds (days)	15 (12–21)	19 (14–26)	13 (10–7)
HMF introduced (mL/kg enteral intake)	118.9, 35.6	109.7, 38.1	126.9, 31.3
Days on parenteral nutrition (days)	16 (11–25)	23 (16–30)	12 (8–7)
Days on intravenous lipid (days)	11 (7–16)	16 (10–23)	9 (5–12)
Proportion of energy from parenteral nutrition (percentage)			
Week 1	95 (82–100)	98 (90–100)	92 (72–100)
Week 2	48 (2–80)	63 (37–87)	21 (0–70)
Human milk as a proportion of measured enteral intake (number of infants)			
>80%	56 (61)	25 (57)	31 (65)
20% to 80%	29 (32)	15 (34)	14 (29)
<20%	7 (8)	4 (9)	3 (6)

Data are presented as *n* (%), Mean, SD and median (interquartile range). Abbreviations: CPAP, continuous positive airway pressure; EDD, estimated due date; HMF, human milk fortifier; ^†^ As defined by requirement for supplemental oxygen at 36 weeks post-menstrual age; ^‡^ *n* = 91 infants; ^§^ To discharge home or estimated due date, whichever came first; * Weight gain from when birth weight regained and maintained 2 days.

**Table 3:** Days 0–3 and weekly intakes of energy, protein and fluid compared with recommendations.

	Energy Intake (kcal/kg/day)	Protein Intake (g/kg/day)		Fluid Intake ^†^ (mL/kg/day)
		Std. Protein	High Protein	
<1000 g (*n* = 44)				
Recommendation: Day 0 parenteral RNI [25]	*60–80*	≥2 *		*90–120*
Day 0	8 (5, 13)	0.2 (0.1–0.3)	0.3 (0.0–0.5)	16 (10–28)
Recommendation: Transition combined ^‡^ [25]	*80–100*	≥3.5 *		
Day 1	27 (24, 35)	0.8 (0.6–0.9)	0.8 (0.5–1.2)	58 (49–76)
Day 2	44 (35, 53)	1.0 (0.7–1.4)	1.4 (1.0–1.7)	85 (75–105)
Day 3	54 (47, 63)	1.6 (1.4–2.0)	2.0 (1.2–2.3)	111 (97–130)
Recommendation: Combined intake ^‡^ [11]	*110–130*	*4.0–4.5*		*135–200*
Week 2 ^§^	97 (76–116)	3.0 (2.0–3.7)	2.8 (2.3–3.5)	152 (124–172)
Week 3	119 (91–142)	3.6 (2.6–4.1)	3.7 (2.7–4.6)	161 (142–170)
Week 4	137 (121–151)	3.6 (3.3–3.9)	4.3 (3.8–4.7)	165 (152–172)
≥1000g (*n* = 48)				
Recommendation: Day 0 parenteral RNI [25]	*60–80*	≥2 *		*70–90*
Day 0	8 (5–15)	0.3 (0.2–0.4)	0.3 (0.0–0.4)	19 (11–34)
Recommendation: Transition combined ^‡^ [25]	*80–100*	≥3.5 *		
Day 1	29 (24–35)	0.8 (0.4–1.1)	1.0 (0.8–1.2)	62 (51–70)
Day 2	44 (34–53)	1.4 (0.8–1.9)	1.3 (1.1–1.7)	85 (66–99)
Day 3	65 (47–72)	2.2 (1.3–2.8)	2.0 (1.5–2.5)	115 (87–127)
Recommendation: Enteral intake ^‡^ [25]	*110–130*	*3.5–4.5*		*135–200*
Week 2 ^§^	120 (96–138)	3.7 (3.0–4.0)	4.3 (2.8–4.8)	164 (149–172)
Week 3	135 (118–147)	3.8 (3.4–4.1)	4.4 (4.0–4.8)	162 (153–172)
Week 4	137 (124–151)	3.8 (3.5–4.1)	4.1 (3.6–4.5)	164 (151–172)

Data are presented as median (interquartile range); Recommended intakes are in italics. * amino acid requirement in g of protein equivalent; ^†^ Includes nutritional fluids only; ^‡^ Intakes are presented as combined enteral and parenteral intakes as while parenteral intakes are titrated down, enteral intakes are correspondingly incrementally increased on an individual basis; ^§^ Study fortifier was begun at median 13 days of age (week of life 2–3) Abbreviations: RNI, recommended nutrient intake.

## Supplementary Materials

The following are available online at http://www.mdpi.com/1660-4601/15/6/1196/s1, Table S1: Clinical characteristics of participants by randomisation group, Table S2: Infant outcomes by randomisation group.
