# Supplementary material for: Dietary Protein Intake, Breast Feeding and Growth in Human Milk Fed Preterm Infants
Source: Int J Environ Res Public Health. 2018 Jun 7;15(6):1196. doi: 10.3390/ijerph15061196 (PMC6025124; doi:10.3390/ijerph15061196)
Supplement: Supplementary file 1 [file ijerph-15-01196-s001.pdf]

Supplementary Material

Table S1 Clinical characteristics of participants by randomisation group

**Infants (*n* = 92)**	**Whole Cohort (n =92)**		**<1000g (n = 44)**		**>=1000g (n = 48)**	
	Std Protein n = 49	Higher Protein n = 43	Std Protein n= 20	Higher Protein n= 24	Std Protein n = 29	Higher Protein n = 19
Gestational age (weeks)	28 (27, 29)	27 (25, 29)	26.5 (25.3, 28)	25.5 (24.3, 27.8)	28 (28, 29)	29 (28, 30)
**Recruitment hospital**						
Women's and Children's Hospital	43	37	18	20	25	17
Flinders Medical Centre	6	6	2	4	4	2
Male infants	21	19	5	14	16	5
**Birth anthropometry**						
Weight (g)	1056, 289	1012, 315	787, 146	789, 165	1242, 203	1294, 219
Length (cm)†	35.6, 3.4(n = 48)	35.7,3.5	32.4, 2.8 (n = 19)	33.4, 2.7	37.7, 1.9	38.6, 1.7
Head circumference (cm)	25.6, 2	25.3, 2.5	23.8, 1.4	23.8, 2.1 (n = 23)	26.8, 1.4	27, 1.6
Caesarean section	32	27	16	15	16	12
Multiple births (number of infants)	20	4	7	3	13	1
**Mothers (n = 80)**	n = 39	n = 41	n = 16	n = 23	n = 23	n = 18
Age (years)	30, 5	29, 6	31, 6	31, 6	30, 5	28, 5
Smoked during pregnancy	6	9	3	5	3	4
Previous preterm birth	5	6	0	1	5	5
Received antenatal steroids	45	36 (n = 42)	15	20	21	15
Received IVF	8	3	2	2	6	1
Completed Secondary Education §	26	31	11	19	15	12

Data are presented as median (interquartile range), *N*, Mean, SD.

Abbreviations: IVF, *in vitro* fertilisation

† *n* = 91 infants

‡Mothers of twins were categorised according to the BW of the first born, if weight categories of the 2 infants differed

§ *n* = 79 mothers

Table S2 Infant outcomes by randomisation group

Clinical Outcomes	Whole cohort (*n* = 92)		<1000g (n = 44)		≥1000g (n = 48)	
	Std Protein n = 49	Higher Protein n = 43	Std Protein n= 20	Higher Protein n= 24	Std Protein n = 29	Higher Protein n = 19
**Chronic lung disease †**	17	20	12	17	5	3
**Received corticosteroid treatment**	17	14	11	12	6	2
**Confirmed necrotizing enterocolitis**	5	3	4	3	1	0
**Intraventricular haemorrhage ‡**	13	10	4	6	9	4
**Sepsis**	7	6	4	4	3	2
**Nasal CPAP**	14	12	3	6	11	6
**Length of stay §**	74, 16	77, 21	84, 12	90, 15	66, 15	61, 16
**Post-menstrual age at discharge or EDD (weeks)**	38 (37, 40)	38 (37, 39)	39 (38, 40)	39 (38, 40)	38 (37, 39)	37 (36, 38)
**Growth outcomes**						
**Weight gain ¶ (g/day)**	28.6, 5.7	29.1, 4.7	26.4, 5	27.4, 4.3	30.1, 5.7	31.3, 4.4
**Intrauterine weight gain (g/kg/day)(26)**						
**Weight gain ¶ (g/kg/day)**	16.3, 1.9	16.6, 2	17, 1.6	16.8, 1.8	15.8, 1.9	16.3, 2.3
**Weight gain before 34 weeks PMA (g/day)**	25.5, 6	24.6, 6.4	22.7, 5.4	22, 5.9	27.5, 5.6	28, 5.5
**Weight gain after 34 weeks PMA (g/day)**	29.3, 6.9	31.3, 6	29, 5.9	2909, 5	29.5, 7.6	33.1, 6.7
**Length gain (cm/week)**	1.1, 0.2	1.2, 0.1	1.1, 0.1	1.2. 0.1	1.1, 0.2	1.2, 0.2
**Head circumference gain (cm/week)**	1, 0.2	1, 0.1	1, 0.1	1, 0.1	1, 0.2	1, 0.2
**Discharge weight (grams)**	2647, 374	2753, 331	2565, 492	2742, 358	2703, 260	2768, 302
**Dietary Outcomes**						
**Age study fortifier commenced (days)**	13 (10, 16)	13 (10, 18)	14 (12, 18)	18 (11, 22)	11 (8, 16)	10 (8, 13)
**Age enteral feeds commenced (days)**	3 (2, 5)	3 (2, 4)	4 (3, 5)	4 3, 5	3 (2, 5)	3 (2, 4)
**Days to reach full enteral feeds (days)**	15 (12, 18)	17 (11, 23)	16 (14, 24)	21 (16, 27)	13 (11, 17)	12 (8, 14)
**HMF introduced (ml/kg enteral intake)**	120 (93, 141)	120 (96, 156)	99 (90, 132)	111 (74, 151)	137 (105, 149)	131 (108, 157)
**Days on parenteral nutrition (days)**	16 (12, 29)	17 (10, 25)	24 (15, 31)	32 (16, 27)	14 (9, 17)	11 (7, 15)
**Days on intravenous lipid (days)**	12 (8, 19)	11 (7, 16)	15 (10, 23)	16 (10, 23)	10 (6, 14)	7 (4, 10)
**Human milk as a proportion of measured enteral intake (number of infants)**						
**>80%**	23	26	8	14	15	12
**20% to 80%**	23	11	10	6	13	5
**<20%**	3	6	2	4	1	2

Data are presented as *N*, Mean, SD and median (interquartile range).

Abbreviations: CPAP, continuous positive airway pressure; EDD, estimated due date; HMF, human milk fortifier

† As defined by requirement for supplemental oxygen at 36 weeks post-menstrual age; **‡** *n* = 91 infants; §To discharge home or estimated due date, whichever came first.; ¶ **Weight gain from when birth weight regained and maintained 2 days**
